# EGFR inhibition prevents in vitro tumor growth of salivary adenoid cystic carcinoma

**DOI:** 10.1186/1471-2121-14-13

**Published:** 2013-03-09

**Authors:** Yi Huang, Tao Yu, Xiaoyue Fu, Jiao Chen, Ying Liu, Chunjie Li, Yichao Xia, Zhuoyuan Zhang, Longjiang Li

**Affiliations:** 1 Department of Head and Neck Oncology, West China College of Stomatology, Sichuan University, No.14, Section 3, Ren Min Nan Road, Chengdu, 610041, China; 2 State Key Laboratory of Oral Diseases, West China Collegel of Stomatology, Sichuan University, Chengdu, China; 3 Department of Thoracic Cancer, West China Hospital of Sichuan University, Chengdu, China; 4 Department of Head and Neck Oncology, Sichuan Cancer Hospital, No. 55, Sec. 4, Renminnan Road, Chengdu, Sichuan, 610041, People’s Republic of China

**Keywords:** Epidermal growth factor receptor (EGFR), Adenoid cystic carcinoma (ACC), Nimotuzumab, Monoclonal antibody, Matrix metalloproteinase (MMP), Epithelial- mesenchymal transition (EMT), Invasion, Cancer therapy

## Abstract

**Background:**

Epidermal growth factor receptor (EGFR) is involved in the development of many human malignant tumors and plays an important role in tumor growth and metastasis. Antagonists of EGFR can suppress the growth of several malignancies; however, their therapeutic effect in adenoid cystic carcinoma (ACC) is controversial.

**Results:**

The increased proliferation of two ACC cell lines induced by EGF-treatment was reversed by nimotuzumab. Regardless of EGF stimulation, nimotuzumab-treated ACC cells were arrested in G1 phase and showed decreased expression of Ki67. In addition, EGF activated the MAPK-dependent pathway and up-regulated the expression of matrix metalloproteinase-9 and Snail, enhancing the invasive potential of an ACC cell line (ACC-M). The effects of EGF were down-regulated by nimotuzumab treatment.

**Conclusions:**

These results suggest that nimotuzumab can inhibit the growth and invasion of ACC cells induced by EGF, probably through inactivation of ERK phosphorylation. Thus, nimotuzumab should be considered as a promising novel agent for the treatment of ACC.

## Background

Adenoid cystic carcinoma (ACC) is a common salivary gland cancer subtype accounting for 22% of salivary gland malignancies and 1% of all head and neck cancers [1]. Perineural invasion, delayed onset of hematogenous metastasis and poor response to traditional chemotherapies are characteristics of this cancer. Local recurrence and distant metastasis, in which extracellular matrix (ECM) and basement membrane degradation are crucial, are responsible for treatment failure. Currently, no systemic therapy is available that effectively inhibits ACC progression. Therefore, further understanding of the development and progression of ACC and identification of new molecular targets are of great importance.

Epidermal growth factor receptor (EGFR) is a member of the ErbB receptor tyrosine kinase family, and upon interacting with its ligand, it undergoes dimerization and autophosphorylation that trigger downstream signaling cascades, such as phosphatidylinositol-3-kinase/Akt and MAPK activation. This signaling pathway enhances tumor cell proliferation, invasion, metastasis, and survival. It is well recognized that matrix metalloproteinase-9 (MMP9), which is closely associated with tumor invasion and metastasis in several human tumors, can be inhibited by EGFR signaling pathway blockade [2-4]. Therefore, the EGFR signaling pathway is believed to play a vital role in the progression and metastasis of tumors, including ACC. Tyrosine kinase inhibitors (TKIs) were first used to block EGFR-mediated signaling pathways. Regretfully, several studies implicated that TKI treatment alone cannot achieve impressive clinical outcomes, possibly because of the kinase-independent functions of EGFR [5]. However, accumulating evidence indicates that nimotuzumab, a humanized neutralizing G1 monoclonal antibody, can suppress cultured epithelial cancer cell proliferation by causing cell cycle arrest. When this antibody binds to the extracellular domain of EGFR, it strongly inhibits EGFR-dependent cellular transformation [6]. Although anticancer activity in advanced squamous cell carcinoma of nimotuzumab has been previously reported [7,8], its role in ACC progression and metastasis is still unclear.

In this study, we evaluated the effects of nimotuzumab on the growth and invasion of ACC-2 and ACC-M, using a tongue squamous cell carcinoma cell line (Tca8113) as a positive control, and analyzed the possible downstream molecular targets and signaling pathway [9-15].

## Results

### Nimotuzumab inhibits cell proliferation *in vitro*

**Figure 1:**
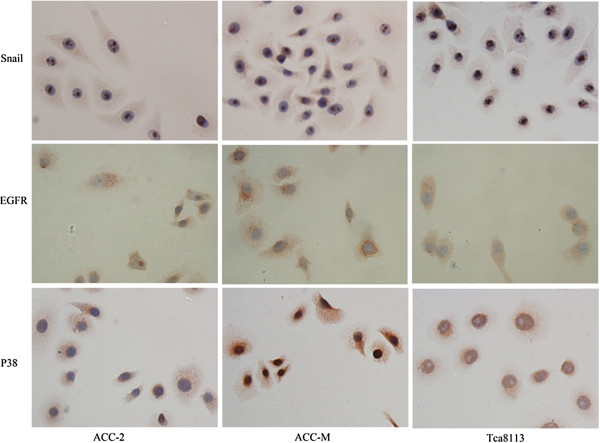
**EGFR**, **P38**, **and Snail expression in ACCs and Tca8113 cell lines.** Immunohistochemistry revealed that EGFR, P38 and Snail are expressed in ACC-2, ACC-M and Tca8113, respectively. Snail exhibited preferences for nuclei, otherwise, both EGFR and P38 exhibited preferences for the cytoplasm and cytomembrane. Magnification, ×400.

Immunohistochemistry was performed to measure the expression of EGFR, MAPK, and Snail in three cell lines. Both EGFR and P38 immunoreactivity were observed mostly in the cytomembrane and cytoplasm, while Snail expression was observed in cell nuclei. Positive staining of three proteins was observed in all three cell lines (Figure 1).

**Figure 2:**
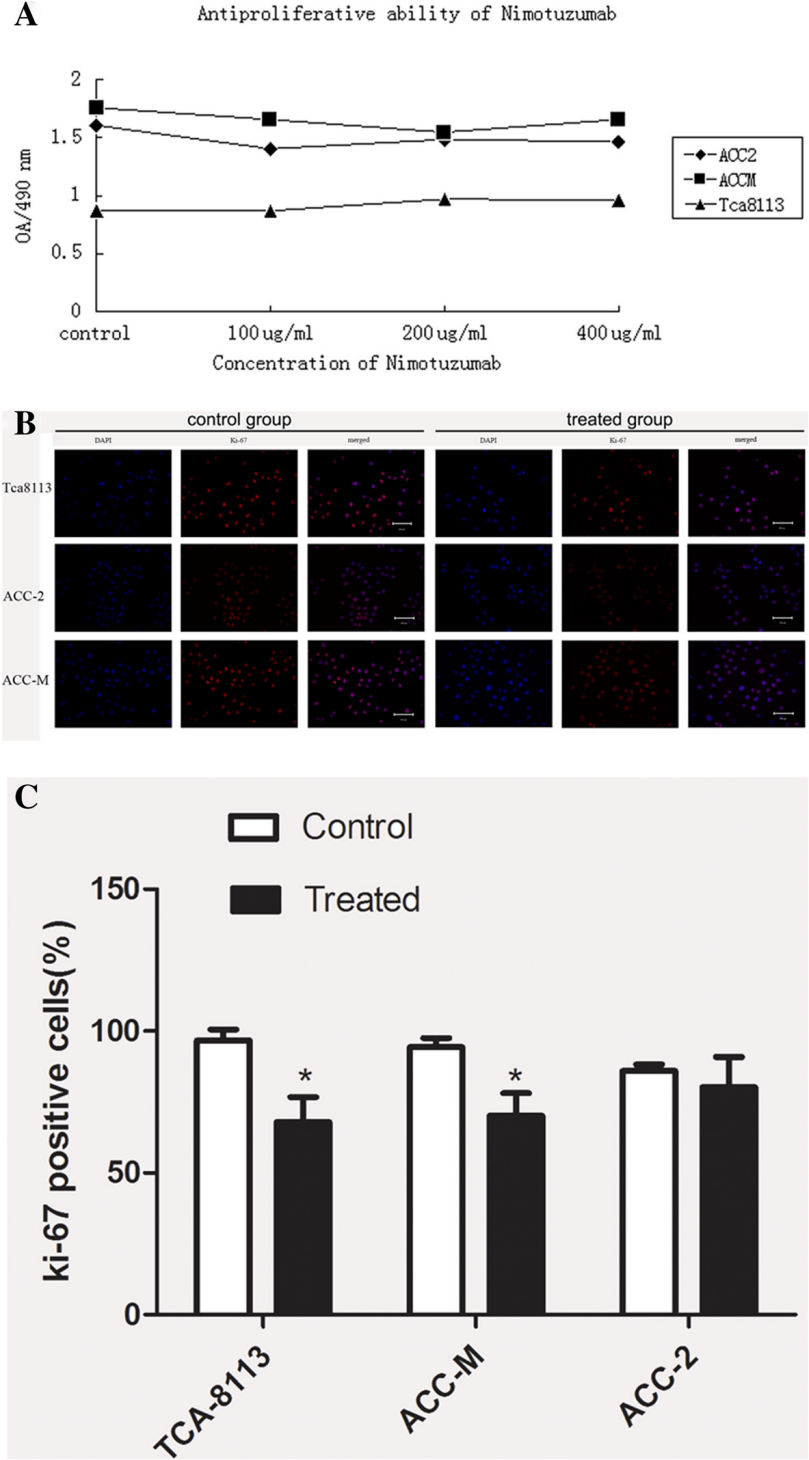
**Nimotuzumab inhibits the Tca8113 and ACC cell proliferation. A**: After treated with different concentration antibody in Tca8113 and ACC cells the growth curve was drawn through CCK-8 assay. The anti-proliferative effect peaked at 100, 200, and 100 μg/ml in ACC-2, ACC-M, and Tca8113 cells, respectively. **B**: The anti-proliferation was also evaluated by by Ki-67 immunostaining. Red fluorescence was reduced in all three cell lines, specifically in Tca8113 and ACC-M cells. Magnification, ×200. **C**: Quantitative analysis of positive Ki-67 Tca8113 and ACCs cells in different groups.

The growth curves showed that the inhibition of proliferation plateaued at doses of 100, 200, and 100 μg/ml nimotuzumab in ACC-2, ACC-M, and Tca8113 cells, respectively. These concentrations of nimotuzumab were used for all subsequent assays (Figure 2A).

Cells were immunolabeled with TRITC-labeled Ki-67 (red fluorescence) and the cell nuclei were stained with DAPI (blue fluorescence). Ki-67-positive cells were defined as actively proliferating cells. The percentage of Ki-67-positive cells of total DAPI-positive cells was 96.7%, 94.3% and 86% in Tca8113, ACC-M and ACC-2 cells, respectively. However, this percentage decreased to 67.9%, 70.2% and 80.3% in all three nimotuzumab-treated cell lines, confirming that nimotuzumab can suppress cancer cell proliferation *in vitro* (Figure 2B, C).

### Nimotuzumab induced G1 phase arrest in ACC-M and Tca8113 cells

**Figure 3:**
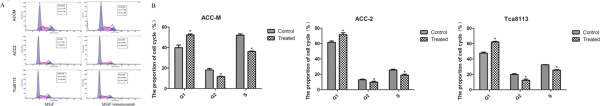
**Effect of nimotuzumab on the cell cycle distribution of Tca8113 and ACC cells.** After treated with medium or medium containing nimotuzumab for 90 min followed by treatment with hEGF (40 ng/ml) for 15 min, cell nuclei were fixed, stained with PI, and analyzed by flow cytometry. Cells were subdivided into two groups: hEGF group and hEGF+ nimotuzumab group. A: Representative histograms are shown that combination treatment resulted in a 9.9% and 5% accumulation of ACC-M and Tca8113 cells in G1 phase. B: The statistical results of cell cycle distribution of Tca8113 and ACC cells are shown.

To detect the effect of nimotuzumab on ACC cell proliferation, the cell cycle distribution of cells treated with nimotuzumab (100 μg/ml for ACC-2 and Tca8113, 200 μg/ml for ACC-M) and/or hEGF (40 ng/ml) in Tca8113 and ACC cell lines were examined. Combined treatment of ACC-M and Tca8113 cells with nimotuzumab and hEGF resulted in a significant G1 phase arrest accompanied by a reduction of the S phase fraction (Figure 3). After treatment with nimotuzumab and hEGF, the percentages of cells in the G1 phase increased from 41.7% to 51.6% in ACC-M and from 56.8% to 61.55% in Tca8113cells. In the absence of hEGF stimulation, nimotuzumab did not significantly affect the cell cycle distribution (data not shown). This confirmed the hypothesis that combined nimotuzumab and hEGF treatment could suppress hEGF-induced cell proliferation.

### Nimotuzumab inhibits EGFR and its downstream molecules

**Figure 4:**
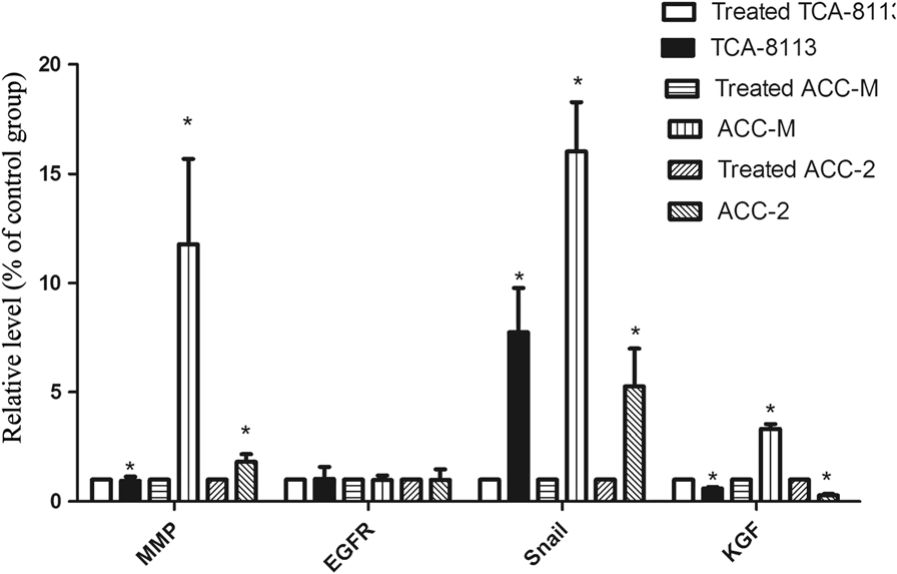
**MMP9**, **EGFR**, **Snail**, **and KGF transcript levels in control and nimotuzumab**-**treated cells.** mRNA levels of MMP9, EGFR, Snail, and KGF were measured by quantitative real-time RT-PCR, normalized against GAPDH, and the indicated% induction or reduction was compared with those in cells cocultured with nimotuzumab. Although, the level of EGFR remained same, MMP9 and Snail expression in Tca8113 and ACC cells was inhibited by nimotuzumab. Error bars indicate SDs, n=3. *, P<0.05.

Serum-starved cells were incubated in medium (control) or medium containing nimotuzumab for 72 h. qRT-PCR analysis demonstrated that Snail mRNA levels were 7.7 ± 2-, 5.25 ± 1.7-, and 16 ± 2.2-fold higher in untreated Tca8113, ACC-2 and ACC-M cell lines than in their treated counterparts, respectively. Conclusively, EGFR mRNA levels were increased but without statistical significance in all three cell lines. Keratinocyte Growth Factor (KGF) mRNA levels were down-regulated by nimotuzumab in Tca8113 and ACC-2 cells and up-regulated in ACC-M cells. EGFR mRNA levels were 2.35 ± 0.35-, 3 ± 0.48-, and 4.3 ± 3-fold higher in untreated Tca8113, ACC-2, and ACC-M cells than in their treated counterparts, respectively. KGF mRNA levels were 0.6 ± 0.07-, 0.28 ± 0.07-, and 3.3 ± 0.22-fold higher in untreated Tca8113, ACC-2, and ACC-M cells than in their treated counterparts, respectively (Figure 4). P38 mRNA levels were not affected by treatment in any cell line observed (data not shown).

**Figure 5:**
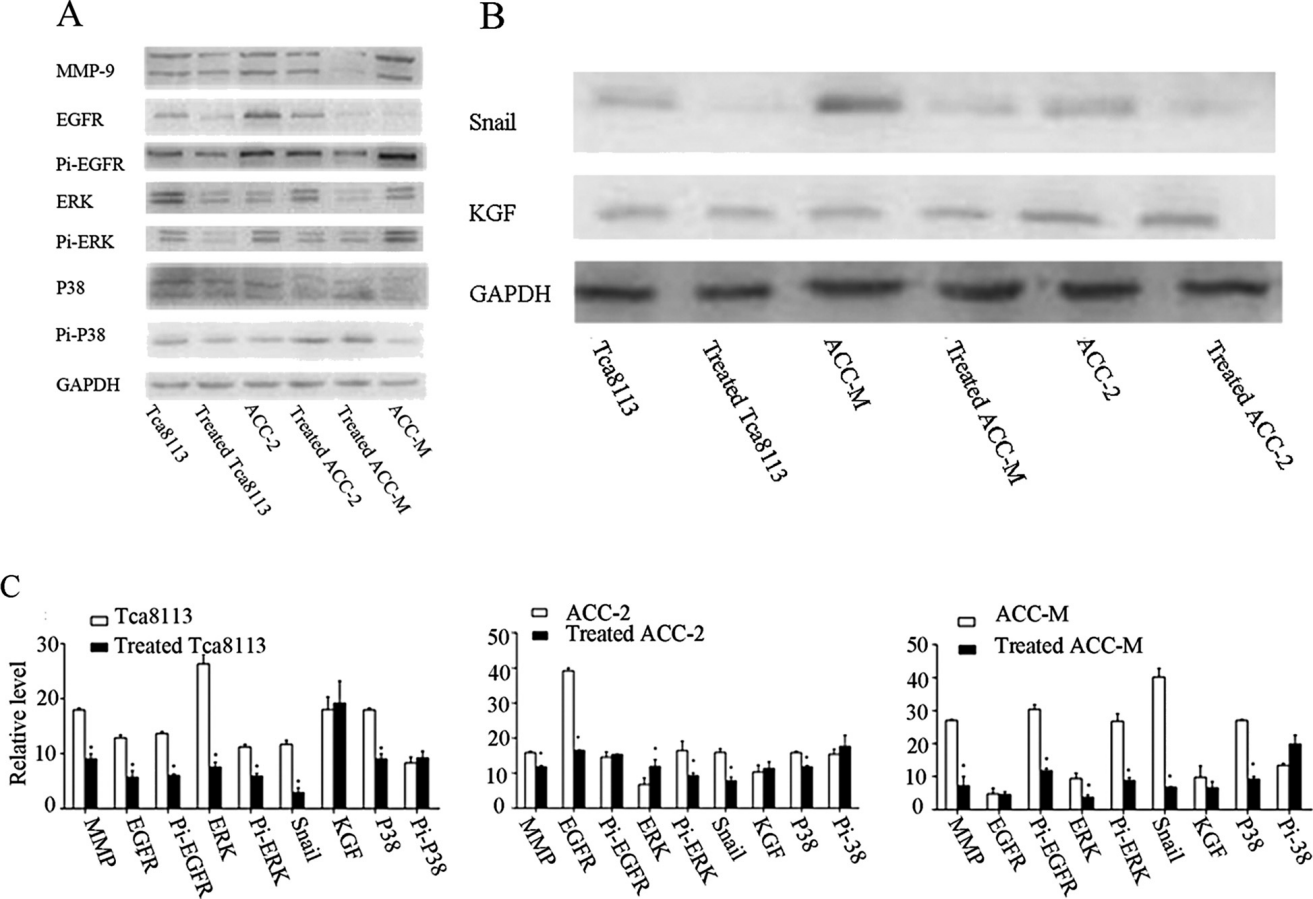
**Nimotuzumab influences the expression of EGFR and its downstream molecules. A**, **B**: Protein level of MMP-9, EGFR, pEGFR, ERK, pERK, Snail, KGF, P-38, and pP38 in control and nimotuzumab- treated group were measured by Western blot. The GAPDH levels were used as the internal controls. **C**: Densitometric analysis of the expression level of the same proteins relative to GAPDH expression. Error bars indicate mean ± SD; n = 3 experiments; *P < 0.05.

The protein levels of EGFR and its downstream molecules were assessed by western blotting. In the first set of studies, we determined whether the *in vitro* exposure of cells to nimotuzumab decreased pEGFR protein expression. Phosphorylated EGFR protein levels were significantly decreased in nimotuzumab-treated Tca8113 and ACC-M cell lines compared with untreated cells. For ACC-2, the phosphorylated EGFR expression level was unchanged (Figure 5A, C). Interestingly, EGFR protein levels were down-regulated whole in the three cell lines. It was reported that EGF activates ERKs mainly through the Grb-2-SOS-Ras-Raf-MEK-ERK pathway [16]. Consistent with previous findings, EGFR signaling blockade significantly decreased ERK and pERK production in Tca8113 and ACC-M cells (Figure 5A, C) [17]. pERK and ERK protein levels were decreased and increased by nimotuzumab treatment in ACC-2 cells, respectively. We also examined P38 expression and found that phosphorylated p38 did not change significantly in all three cell lines regardless of nimotuzumab treatment. Although nimotuzumab suppressed Snail expression in all three cell lines, it did not affect KGF expression in these cells (Figure 5B, C).

**Figure 6:**
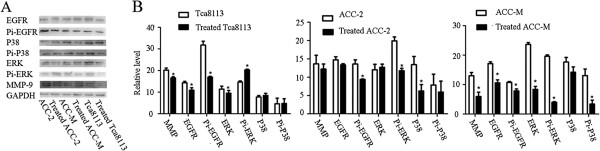
**Nimotuzumab impeded the EGF**-**EGFR interaction. A**: Serum-starved cells either were treated with medium (control) or medium containing nimotuzumab for 90 min followed by treatment with hEGF (40 ng/ml) for 15 min. Protein level of MMP-9, EGFR, pEGFR, ERK, and pERK were assessed by Western blotting. GAPDH expression is also shown. **B**: Densitometric analysis of the expression of the same proteins relative to GAPDH expression. Error bars indicate mean ± SD; n = 3 experiments; *P < 0.05.

To evaluate the proposed mechanism of action of nimotuzumab *in vitro*, we studied its ability to inhibit EGF-induced tyrosine phosphorylation of EGFR and MAPK by western blotting. Serum-starved cells were treated with medium (control) or medium containing nimotuzumab for 90 min followed by treatment with hEGF (40 ng/ml) for 15 min. In agreement with previous findings, besides decreased EGFR protein levels, EGF-induced EGFR activation was abrogated by nimotuzumab treatment in ACC-M and ACC-2 cell lines, resulting in pERK down-regulation [18]. Unexpectedly, although ERK expression was decreased in ligand-stimulated Tca8113 cells, its active form was increased. No significant differences were found in P38 and pP38 expression in all three cell lines (Figure 6A, B).

### Nimotuzumab inhibits cell invasion

**Figure 7:**
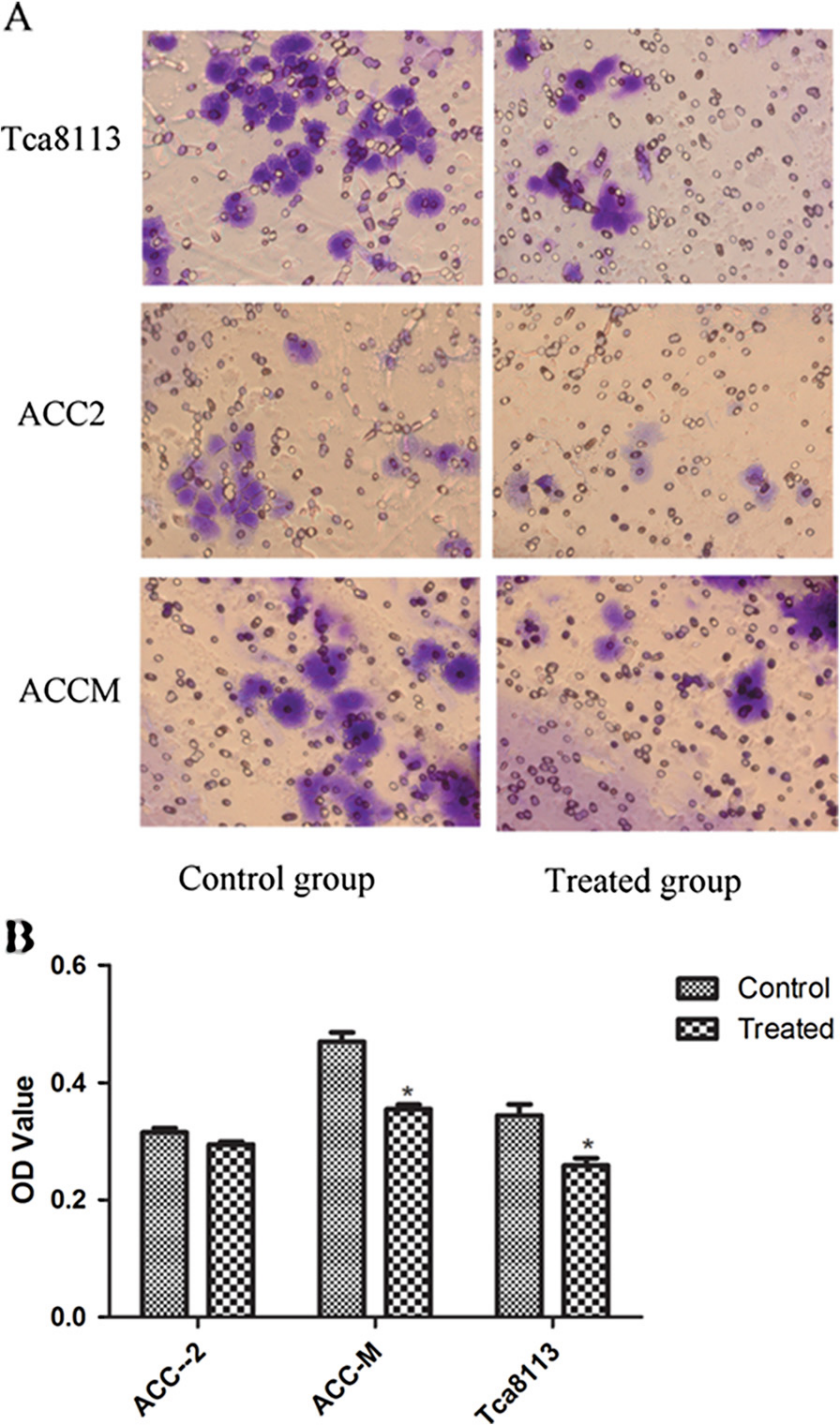
**Nimotuzumab inhibits cell invasion. A**: Starved cells were allowed to invade the membrane for 72 h in the presence or absence of nimotuzumab followed by the treatment with hEGF. Tca8113 and ACC-M cell invasive ability were markedly inhibited by nimotuzumab. Magnification,×200. **B**: The quantitative analysis of invaded cells, which were eluted using 10% acetic acid, and the OD value at 570 nm.

To investigate whether nimotuzumab could suppress the invasive phenotypes of Tca8113 and ACC cells *in vitro*, we conducted invasion assays using Matrigel-coated Transwell chambers. All three cell lines could migrate across the membrane. Among them Tca8113 and ACC-2 cells exhibited the highest and lowest capacity to migrate, respectively. Pure cultured or co-cultured cells on the upper chamber were allowed to migrate for 72 h followed by treatment with hEGF. Reduced invasive ability of Tca8113 was observed. The same tendency was found in the ACC-M cell line (Figure 7A). The optical density (OD) of the stained cells was measured at 570 nm (Figure 7B). To confirm whether nimotuzumab regulated cell invasion by modulating MMP9 expression, which is closely associated with tumor invasion and can be inhibited by EGFR signaling blockade, qRT-PCR and western blot analyses were performed. MMP9 mRNA and protein levels were decreased significantly by nimotuzumab treatment in all three cell lines (Figures 4, 5A, 6A).

## Discussion

ACC preferentially metastasizes to the lungs, bone, liver, and brain, unlike squamous cell head and neck cancer (SCCHN) [19]. Local and distant recurrence can occur concurrently irrespective of initial local control, suggesting that distant and local recurrence is a separate disease [20,21]. ACC is typically controlled through surgery and postoperative adjuvant radiotherapy. Chemotherapy has also been attempted with extremely variable and inconsistent outcomes [22]. To date, the most effective chemotherapy treatment is the combination of cyclophosphamide plus doxorubicin and cisplatin [23]. Vascular EGFR (VEGFR)-targeted therapies have been suggested to increase tumor invasiveness. VEGFR and EGFR signaling pathways appear to be closely related, casting doubt on the effectiveness of anti-EGFR therapy [24,25]. Furthermore, the efficacy of small-molecule tyrosine kinase inhibitors including gefitinib, imatinib, and lapatinib has also been investigated with disappointing results [23]. Therefore, new therapeutic regimens should be developed to treat advanced ACC. Many preclinical studies suggest that nimotuzumab can be used as part of a multimodality treatment scheme. Combined radiation therapy and EGFR inhibition is considered a standard concept for SCCHN [26]. Targeted therapies such as nimotuzumab have also attracted attention for the treatment of ACC. In the present study, nimotuzumab inhibited tumor cell proliferation *in vitro* via an EGFR-dependent signaling transduction mechanism.

To facilitate cell motility, the invading cells must alter their cell-cell adhesion properties and the ECM environment. In this study, we observed that Tca8113, ACC-2 and ACC-M cell lines expressed MMP9, Snail, P38 and EGFR in accordance with previous findings [27]. Unlike vandetanib, which suppresses cell growth via a direct pro-apoptotic mechanism, nimotuzumab inhibited cellular proliferation as confirmed by CCK-8 assay and Ki-67 immunofluorescence [18,25]. Nimotuzumab inhibited the phosphorylation of EGFR and ERK, two downstream effectors of the EGFR signaling pathway that promote cellular proliferation and invasive potential. Furthermore, nimotuzumab down-regulated the mRNA and protein expression of Snail, which can mediate EMT. We hypothesized that binding of nimotuzumab to EGFR impeded the interaction between EGF and EGFR, thus inhibiting the activation of downstream cell signals and preventing increased cellular proliferation and invasive potential. P38 protein levels were not altered by nimotuzumab in the nimotuzumab group or combination group, suggesting that the inhibitory function of this antibody did not involve P38 MAPK signaling.

Interestingly, EGFR mRNA in the experimental group increased but was not statistically significant, compared with the control group. However, EGFR protein levels were significantly down-regulated in both the nimotuzumab group and combination group. We hypothesized that the down-regulation was caused by nimotuzumab blocking the binding site for the antibody used for western blotting.

## Conclusions

Two unique features of ACCs are a predilection for perineural infiltration and a low incidence of lymph node metastasis. KGF expression was not affected by nimotuzumab treatment. Thus, the predilection of ACC for perineural invasion may be explained by the activity of other adhesion-related molecules.

MMPs facilitate cancer cell invasion and metastasis by degrading ECM proteins. Under normal conditions, MMP9 expression is low or absent in ACC cells. However, the EGFR signaling pathway has been implicated in MMP9 up-regulation in SCCHN [27]. In this study, MMP9 was expressed in both carcinoma cell lines. Its expression was inhibited by nimotuzumab treatment. As tumor cell invasion is MMP-dependent, MMPs likely participate in the antitumor effects of nimotuzumab.

In summary, we demonstrated that nimotuzumab could inhibit the growth and invasion of ACC cell lines induced by EGF, probably through the inactivation of ERK phosphorylation, suggesting that the *in vivo* efficacy of nimotuzumab against metastatic ACC should be examined. Nimotuzumab should be considered as a promising novel agent for the treatment of ACC.

## Methods

### Cell culture

ACC-2, ACC-M and Tca8113 cells were provided by the State Key Laboratory of Oral Diseases, Sichuan University. Cells were cultured with RPMI 1640 medium supplemented with 10% FBS, penicillin G (100 U/ml), and streptomycin (100 μg/ml) at 37°C in a humidified atmosphere of 5% CO_2._

### Antibodies and reagents

Nimotuzumab was obtained from Biotech Pharma and freshly diluted in serum-free RPMI 1640 to the designed concentration for each experiment. Cell Counting Kit-8 (CCK-8) was obtained from Dojindo Laboratories. The peroxidase-labeled secondary antibody and ECL system were obtained from Millipore. Anti-phospho-EGFR, anti-KGF, and anti-Slug primary antibodies were obtained from Abcam. Anti-Snail, anti-P38/pP38, anti-ERK/pERK, and hEGF were purchased from Cell Signaling Technology. Anti-MMP-9 and mouse anti-Ki67 were purchased from Zhongshan Goldenbridge Biotechnology. Antibodies to EGFR, SNAI 1, and P38 were obtained from Bioworld Technology. Biotinylated secondary antibody, the horseradish peroxidase (HRP)-streptavidin complex, and 3,3-diaminobenzidine were obtained from ZSGB-BIO.

### Cell proliferation assay

Cell proliferation assays were performed using CCK-8. After exposure to different concentrations of nimotuzumab (0–400 μg/ml) for 72 h, the cells were reseeded in 96-well plates at a density of 3×10^3^ cells/well for 24 h. The experimental group was subdivided into four groups according to the nimotuzumab concentration: 400, 200, 100, and 50 μg/ml. Culture medium containing 10% FBS was used as a negative control. After treatment, 10 μl of CCK-8 solution were added to each well, followed by a 2-h incubation. The optical density (OD) at 490 nm was measured to estimate the number of living cells (Thermo Scientific Varioskan Flash).

### Immunohistochemistry and immunofluorescence

Cells grown on coverslips in the presence of nimotuzumab for 72 h were fixed with 4% paraformaldehyde for 15 min. The cells were further treated with 0.5% Triton X-100 for 15 min for intracellular staining in the case of Snail and Ki67.

Cells on the coverslips were stained using the standard avidin-biotin complex procedure. After 0.3% hydrogen peroxide was used to block endogenous peroxidase, slices were blocked with goat serum. After separate incubations with rabbit polyclonal anti-EGFR, anti-SNAI1, and anti-P38, the slices were incubated with biotinylated secondary antibody and the HRP-streptavidin complex for 30 min at 37°C. Finally, the sections were stained with 3,3-diaminobenzidine and counterstained with hematoxylin. Goat serum was used as an isotype control. Brown particles in the cytoplasm or nucleus indicated positive expression.

The immunofluorescence of Ki-67 was assayed using the 1:100-diluted mouse anti-Ki67 antibody. The samples were then washed and incubated with Alexa Fluor 594 goat anti-mouse IgM (Invitrogen, USA) for 1 h at room temperature in the dark. The nuclei were visualized using DAPI (Sigma). Samples were photographed with an AxioCamMR monochrome digital camera mounted on a Zeiss AxioImager Z1 microscope equipped with Zeiss AxioVision 4 software (Zeiss, Nürnberg, Germany).

### Quantitative real-time PCR

Total RNA was extracted from cells using Trizol reagent (Invitrogen Life Technologies, Gaithersburg, MD) according to the manufacturer’s instructions. Total RNA (500 ng) was reverse-transcribed using the TaKaRa Prime Script RT Reagent kit following the manufacturer’s protocol (37°C for 15 min, 85°C for 5 s). All primers sequences are shown Table 1. qRT-PCR was performed using the Applied Biosystems 7300 Real Time PCR System and SYBR Premix Ex Taq II™ system (TaKaRa). The thermal cycling conditions were as follows: initial denaturing at 95°C for 30 s, followed by 40 cycles of denaturing at 95°C for 5 s and annealing at 60°C for 31 s. The housekeeping gene GAPDH was used as an internal control. The relative quantification was given by the threshold cycle (C_t_) values, determined for triplicate reactions for the experimental and control groups. The relative gene expression level was calculated using the 2^-△△Ct^ method.

**Table 1:** The primers for quantitative real-time RT-PCR

**Gene**	**Forward Primer (5'-3')**	**Reverse Primer (5'-3')**
KGF	AAAAGAGGCAAAGTAAAAGGGAC	CCATTTAGCTGATGC ATATGTGTTG
Snail	CAAGGATCTCCAGGCTCGAA	GGCACTGGTACTTCTTGAC
EGFR	GAGTAACAAGCTCACGCAGTTG	GAGGACATAACCAGCCACCTC
GAPDH	CTTTGGTATCGTGGAAGGACTC	GTAGAGGCAGGGATGATGTTCT

### Western blot analysis and densitometry

After a 30-min lysis using lysis buffer (50 mM Tris–HCl, pH 8.0; 5 mM EDTA; 150 mM NaCl; 0.5% Nonidet P-40; 0.5 mM PMSF; 0.5 mM DTT), the supernatant was collected by centrifugation at 14,000 rpm for 15 min. Approximately 50 μg of total protein from the supernatant, as determined using the BCA protein assay kit (keyGEN bioTECH, China), were resolved on a 10% SDS-PAGE gel before being transferred to a PVDF membrane using semi-dry (BioRad, Hercules, CA) and wet transfer systems (BioRad mini-protean tetra cell). Membranes were blocked in Tris-buffered saline (TBS: 150 mM NaCl; 50 mM Tris, pH 7.4) containing 5% fat-free dried milk for 2 h and then incubated with anti-EGFR/pEGFR, anti-KGF, anti-Snail, anti-P38/pP38, anti-ERK/pERK, anti-MMP-9, or anti-GADPH primary antibody overnight. After incubation with diluted HRP-conjugated goat anti-rabbit and goat anti-mouse secondary antibodies for 60 min at room temperature, membranes were washed in TBS containing 0.1% Tween-20. Signals were visualized using the EasyECL Western blot kit, and images were captured using a Chemidoc XRS (BioRad). Densitometry was performed using Quantity One software (BioRad, Hercules, CA).

### Flow cytometry

The effect of nimotuzumab on cell cycle distribution was determined by flow cytometry. Cells were starved in serum-free medium for 24 h and then incubated with diluted hEGF and/or nimotuzumab. Untreated cells were used as the negative control. Cells were washed with PBS and fixed in 70% ethanol overnight at 4°C, followed by treatment with 80 mg/ml RNaseA and 50 mg/ml PI for 30 min and analysis using a Beckman FC500.

### Cell invasion assay

The Transwell assay was performed using the QCM™ 24-well Invasion Assay kit (Chemicon International, USA), based on the Boyden chamber principle. Starved cells were resuspended in serum-free RPMI 1640 containing appropriate concentrations of nimotuzumab, and 1×10^4^ cells were added to the upper chamber. Five hundred microliters of culture medium supplemented with 10% FBS were added to the lower chamber as a chemoattractant. The cell culture plates were incubated for 72 h. After scraping off noninvaded cells and the ECM gel from the interior of the insert with a cotton swab, the polycarbonate membrane was stained with 0.1% crystal violet. Acetic acid (10%) was used to dissolve stained cells, and the eluent was quantified by measuring the OD at 570 nm.

### Data statistics

All quantified data represent an average of at least three samples. Data are expressed as the mean ± standard deviation (SD). Error bars represent the SD. Statistical analysis was performed using Student’s *t*-test, and P<0.05 indicated significant differences.

## Abbreviations

ACC: Adenoid cystic carcinoma; CCK-8: Cell proliferation assay kit; ECM: Extracellular matrix; EGFR: Epidermal growth factor receptor; EMT: Mesenchymal transitions; GAPDH: Glyceraldehyde-3-phosphate dehydrogenase; ACC-M: High metastasis cell line of salivary gland adenoid cystic carcinoma; KGF: Keratinocyte growth factor; MAPK: Mitogen-activated protein kinase; MMP9: Matrix metalloproteinase 9; qRT-PCR: Quantitative real-time RT-PCR; SCCHN: Squamous cell carcinoma of the head and neck; Tca8113: Tongue squamous cell carcinoma cell line

## Competing interests

No authors of this manuscript have any competing interests to disclose.

## Authors’ contributions

YH participated in the design and conduction of experiments, data analysis, and final drafting and writing of the manuscript. TY, XF and JC all contributed new reagents for these experiments. YL and ZZ were involved in research design and contributed to the drafting of the manuscript. LL was closely involved in research design and drafting of the final manuscript. All authors read and approved the final manuscript.
